# ^V600E^BRAF Inhibition Induces Cytoprotective Autophagy through AMPK in Thyroid Cancer Cells

**DOI:** 10.3390/ijms22116033

**Published:** 2021-06-03

**Authors:** Eva Jiménez-Mora, Beatriz Gallego, Sergio Díaz-Gago, Marina Lasa, Pablo Baquero, Antonio Chiloeches

**Affiliations:** 1 Departamento de Biología de Sistemas, Unidad de Bioquímica y Biología Molecular, Facultad de Medicina, Campus Universitario, Universidad de Alcalá, Alcalá de Henares, 28871 Madrid, Spain; evamjmora@gmail.com (E.J.-M.); b.gallego@edu.uah.es (B.G.); sergio.diazgago@edu.uah.es (S.D.-G.); pablo.baquero@uah.es (P.B.); 2 Departamento de Bioquímica-Instituto de Investigaciones Biomédicas “Alberto Sols”, Universidad Autónoma de Madrid-Consejo Superior de Investigaciones Científicas, 28029 Madrid, Spain; mlasa@iib.uam.es

**Keywords:** ^V600E^BRAF, autophagy, LKB1, AMPK, ULK1, survival, thyroid cancer

## Abstract

The dysregulation of autophagy is important in the development of many cancers, including thyroid cancer, where ^V600E^BRAF is a main oncogene. Here, we analyse the effect of ^V600E^BRAF inhibition on autophagy, the mechanisms involved in this regulation and the role of autophagy in cell survival of thyroid cancer cells. We reveal that the inhibition of ^V600E^BRAF activity with its specific inhibitor PLX4720 or the depletion of its expression by siRNA induces autophagy in thyroid tumour cells. We show that ^V600E^BRAF downregulation increases LKB1-AMPK signalling and decreases mTOR activity through a MEK/ERK-dependent mechanism. Moreover, we demonstrate that PLX4720 activates ULK1 and increases autophagy through the activation of the AMPK-ULK1 pathway, but not by the inhibition of mTOR. In addition, we find that autophagy blockade decreases cell viability and sensitize thyroid cancer cells to ^V600E^BRAF inhibition by PLX4720 treatment. Finally, we generate a thyroid xenograft model to demonstrate that autophagy inhibition synergistically enhances the anti-proliferative and pro-apoptotic effects of ^V600E^BRAF inhibition in vivo. Collectively, we uncover a new role of AMPK in mediating the induction of cytoprotective autophagy by ^V600E^BRAF inhibition. In addition, these data establish a rationale for designing an integrated therapy targeting ^V600E^BRAF and the LKB1-AMPK-ULK1-autophagy axis for the treatment of ^V600E^BRAF-positive thyroid tumours.

## 1. Introduction

Thyroid cancer is the most common endocrine malignancy [1] and its incidence is continuously increasing worldwide. Based on their histopathological characteristics, thyroid carcinomas are classified into different types, including papillary thyroid cancer (PTC) and anaplastic thyroid cancer (ATC) [2,3]. PTCs are the most frequent, accounting for 80% of all cases. The majority of PTCs, treated with the appropriated therapy, display a good prognosis and are usually curable, with a 5-year survival rate over 95% [4]. However, its recurrence rate is approximately 15%, with a small percentage of PTCs progressing to a more aggressive disease that does not respond to standard treatments [5,6]. By contrast, ATCs represent a low percentage among all thyroid cancers, but they are extremely aggressive, highly resistant to conventional chemotherapy and radiotherapy and show a 1-year survival rate of less than 10% [7,8].

The ^V600E^BRAF mutation has been identified in approximately 7–10% of all human cancers, including melanoma and colorectal cancer, and is one of the most common mutations observed in thyroid cancer, with an average incidence of 45% in PTCs and 25% in ATCs [9]. This mutation leads to a constitutive activation of the mitogen-activated protein kinase kinase (MEK)-extracellular-regulated kinase (ERK) signalling pathway of the mitogen-activated protein kinases (MAPKs), which in turn, results in different hallmarks of cancer; increased proliferation, upregulation of migration, invasion and protection from apoptosis [10,11,12,13,14]. Moreover, the presence of this oncogene is strongly associated with the clinicopathological features of advanced stages of thyroid cancer, such as a rapid progression, extrathyroidal invasion, lymph node metastases and tumour recurrence [9,15]. Therefore, the development of therapeutic strategies targeting ^V600E^BRAF has been promoted in recent years. In line with this, some ^V600E^BRAF inhibitors (BRAFi), such as Vemurafenib and Dabrafenib, alone or in combination with the MEK inhibitor Tramatinib have been recently approved for the treatment of ^V600E^BRAF positive patients with metastatic melanoma or ATC, respectively. Nevertheless, despite the promising improvements in the response rates with these anti-^V600E^BRAF therapies, a significant percentage of patients progress to a more advanced stage of the disease and/or develop resistance to BRAFi. The most common mechanisms involved in BRAFi-resistance include the reactivation of the MEK-ERK pathway caused by the upregulation of tyrosine kinase receptors and activation of other signalling pathways, such as the phosphoinositide 3-kinase (PI3K)-AKT axis [16,17,18]. Indeed, the presence of secondary mutations in the MAPK pathway, such as changes in RAS proteins or concurrent mutations with PI3KCA have been described as important mechanisms of resistance to ^V600E^BRAF inhibition in thyroid cancers [19,20,21,22]. On the other hand, acquired resistance to BRAFi may lead to the induction of autocrine loops that reactivate MAPK and PI3K/AKT pathways in ATC and PTC cells after BRAFi treatment. For instance, it has been reported that the upregulation of neuregulin-1 (NRG1) results in the induction of human epidermal growth factor receptor 3 (HER3)-ligand-dependent activation of HER2/HER3 signalling pathway [23]. Furthermore, ERK signalling reactivation following the overexpression and autocrine activation of the c-Met receptor by HGF was shown to mediate resistance to ^V600E^BRAF inhibition in ATCs [24,25]. Moreover, autocrine interleukin-6 (IL-6) secretion contributes to BRAF-resistance in thyroid cancer cells through the induction of JAK/STAT3 and MAPK signalling [26]. Hence, understanding the underlying mechanisms of resistance to selective BRAFi is essential to develop safe and effective therapeutic approaches for thyroid cancer treatment.

Autophagy is an evolutionarily conserved catabolic process used by cells to recycle cytoplasmic material, misfolded proteins or damaged organelles [27,28]. This process is enabled by the formation of a double membrane vesicle called autophagosome, which in late stages of the process fuses with the lysosome, forming the autolysosome, delivering its content for degradation by lysosomal hydrolytic enzymes [27,28]. Some of the products that result from the degradation stage are metabolites that can return to the cytoplasm, where they can be reused as energetic sources or substrates for biosynthetic reactions that allow cells to maintain cellular homeostasis under nutrient-poor conditions and ensure cell survival after exposure to stress factors [29,30]. In recent years, it has become increasingly evident that autophagy plays an important role in the development of cancer [31,32]. In this sense, in certain cancers, autophagy is necessary for the growth, survival and aggressiveness of tumour cells [33,34]. Additionally, autophagy may also play a role in acquiring resistance to anti-cancer therapies. Thus, many therapeutic regimens induce cytoprotective autophagy, rendering cancer cells less sensitive to these agents [35,36]. Accordingly, genetic and pharmacological inhibition of autophagy disrupt these compensatory effects and, importantly, this fact has led to the use of autophagy inhibitors as sensitizing compounds to chemotherapy/radiotherapy in many types of tumours, providing new therapeutic opportunities [37,38]. To date, autophagy has also been implicated in the BRAFi acquired-resistance in certain tumours [39,40,41,42]; however, its role and relationship with ^V600E^BRAF in thyroid cancer remain controversial. In fact, it has been reported that activation of autophagy by different antitumour treatments sensitizes thyroid cancer cells to chemotherapy and radiotherapy [43,44,45], and the opposite, that blocking the induction of autophagy following Vemurafenib treatment sensitizes ^V600E^BRAF-mutant thyroid cancer cells to this BRAFi [46]. Moreover, different molecular mechanisms have been proposed to explain the activation of autophagy by anticancer agents on cell death and resistance to ^V600E^BRAF inhibition. Among these mechanisms, the regulation of AMP-activated protein kinase (AMPK) and the mechanistic Target Of Rapamycin (mTOR) pathway in colorectal cancer cells (CRC) and the activation of endoplasmic reticulum (ER) stress response in melanoma and thyroid cancer have been described [40,46,47].

In this study, we have further investigated the role of ^V600E^BRAF inhibition on autophagy in thyroid cancer cells, analysed the molecular mechanisms underlying this effect and tested the therapeutic potential of combining autophagy and ^V600E^BRAF inhibitors. Our results demonstrate that pharmacological inhibition of ^V600E^BRAF with the specific BRAFi PLX4720 or silencing of its expression promotes autophagy through activation of the liver kinase B1 (LKB1)—AMPK–Unc-51 Like Autophagy Activating Kinase 1 (ULK1) pathway, which protects against cell death. Consequently, autophagy inhibitors sensitize thyroid cancer cells to ^V600E^BRAF inhibition and enhance the antitumor effects of PLX4720 in vivo, providing a rationale for the development of further studies which combine the blockage of autophagy with selective ^V600E^BRAF inhibitors in thyroid cancer.

## 2. Results

### 2.1. ^V600E^BRAF Inhibition Induces Autophagy in Thyroid Cancer Cells

**Figure 1:**
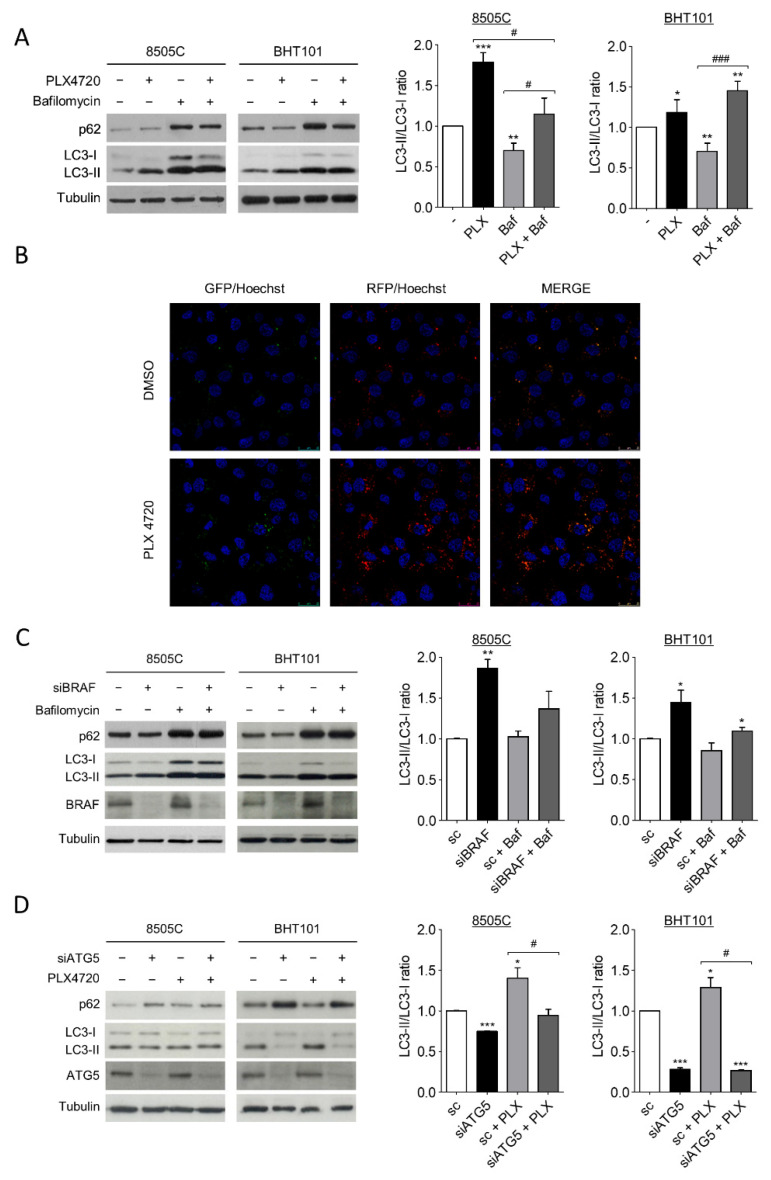
^V600E^BRAF inhibition induces autophagy in thyroid cancer cells. (**A**) LC3 and p62 protein levels (left blots), and quantitative analysis of LC3-II/LC3-I ratios (right graphs) in cells incubated for 24 h with DMSO (-) or PLX4720 (PLX), in the absence or presence of Bafilomycin A1 (Baf). (**B**) Immunofluorescence in 8505C cells transiently transfected with the mRFP/GFP-LC3 (ptfLC3) plasmid for 48 h, and then treated with DMSO or PLX4720 for 24 h. Autophagosomes are identified by yellow puncta (green and red) and autolysosomes by “red only” puncta. Nuclei were labelled with Hoechst dye. Scale bar is 25µm. (**C**) LC3, p62 and BRAF protein levels (left blots), and quantitative analysis of LC3-II/LC3-I ratios (right graphs) in cells transfected with either an oligo control (sc) or specific siRNA for BRAF (siBRAF) for 72 h, and treated with Bafilomycin A1 for the last 24 h. (**D**) LC3, p62 and ATG5 levels (left blots), and quantitative analysis of LC3-II/LC3-I ratios (right graphs) in cells transfected with an oligo control (sc) or with specific siRNA for ATG5 (siATG5) for 48 h, and then left untreated or treated with PLX4720 for 24 h. For each experiment, membranes were reprobed with anti-β-Tubulin as a loading control. Graphic bars represent the LC3-II/LC3-I ratio, calculated after quantitation of LC3-II and LC3-I bands of the blots, and are presented as fold induction relative to the untreated cells. Blots are from one representative experiment and data shown represent the mean ± SEM of the quantitation of at least three independent experiments performed with similar results. Significant differences compared to the corresponding controls: * 0.01 < *p* < 0.05, ** 0.001 < *p* < 0.01, *** *p* < 0.001, compared to untreated cells; ^#^ 0.01 < *p* < 0.05, ^###^ *p* < 0.001, compared to Bafilomycin A1 treatment.

During autophagy, the cytoplasmic form of microtubule-associated protein 1A/1B-light chain 3 (LC-3), LC3-I, is converted into the lipidated and autophagosome membrane-targeted form, LC3-II, which is degraded in autolysosomes during late stage of the process. Then, as previously stablished, the LC3-II/LC3-I ratio is used as a reliable marker of autophagy; in the presence of a lysosomal inhibitor, an increase in LC3-II levels indicates an induction of autophagy [48]. On the other hand, the degradation of autophagy cargo receptor p62 protein levels is another marker of active autophagy. Thus, to investigate the role of ^V600E^BRAF on autophagy in thyroid cancer, we first analysed both LC3-II/LC3-I ratio and p62 levels in 8505C and BHT101 cells after treatment with the ^V600E^BRAF inhibitor, PLX4720. The results in Figure 1A showed that PLX4720 produced an increase in LC3-II/LC3-I ratio in both cell lines, whereas the decrease in p62 was only observed in BHT101 cells (Figure 1A). Further analysis in the presence of the autophagy inhibitor Bafilomycin A1 indicated similar effects, reinforcing the hypothesis that an increase in autophagy flux was induced by PLX4720 (Figure 1A).

To precisely assess autophagy flux, we examined the intracellular location of LC3 in autophagic vesicles by the transfection of a tandem-tagged GFP-mRFP-LC3 plasmid in 8505C cells. This assay enables different stages of autophagy to be visualized by fluorescence microscopy. Thereby, red and green puncta (yellow when merged) represent autophagosomes, whereas red only puncta indicate autolysosomes, as acidic environment in the lysosomes quench the GFP fluorescence [49]. Our results showed that treatment with PLX4720 induced a greater increase in both, the number of autophagosomes (yellow) and autolysosomes (red) compared to control cells (Figure 1B), indicating higher autophagy levels in PLX4720-treated cells.

To rule out possible off-target effects of PLX4720, we next performed similar experiments silencing ^V600E^BRAF expression. The LC3-II/LC3-I ratio was increased in 8505C and BHT101 cells transfected with a specific siRNA targeting BRAF compared to control cells, both in the absence or presence of Bafilomycin A1 (Figure 1C). Similar to our results with PLX4720, in BRAF-knockdown cells, p62 levels were lower than in control cells only in the BHT101 cells, suggesting that p62 regulation was not dependent on ^V600E^BRAF in 8505C cells. As expected, the reduction in p62 levels in cells with reduced ^V600E^BRAF expression was reversed following lysosomal inhibition in Bafilomycin A1-treated cells (Figure 1C).

These data indicate that both the pharmacological and genetic inhibition of ^V600E^BRAF induce autophagy in thyroid cancer cells.

To further study whether the effects on LC3 following ^V600E^BRAF inhibition were due to the canonical autophagy pathway, both the LC3-II/LC3-I ratio and p62 levels were analysed in cells in which the protein autophagy related 5 (ATG5) was knockdown by specific siRNA. ATG5 is part of the conjugation system assembled during autophagosome formation and is therefore an essential factor for its completion. As expected, the reduction of ATG5 expression in 8505C and BHT101 cells led to a decrease in LC3-II/LC3-I ratio and an accumulation in p62 in both cell lines (Figure 1D). Moreover, in the absence of ATG5, the increase in LC3-II/LC3-I ratio achieved by PLX4720 treatment did not occur, demonstrating that ^V600E^BRAF regulates the canonical autophagy pathway. Once again, p62 levels were only reversed by ATG5 knockdown in PLX4720-treated BHT101 cells but not in 8505C cells (Figure 1D). These data evidence that ATG5 abrogation blocks PLX4720-induced autophagy. Altogether, these results demonstrate that ^V600E^BRAF inhibition induces autophagy flux in thyroid cancer cells carrying this mutation.

### 2.2. Inhibition of ^V600E^BRAF Activates the AMPK Pathway in Thyroid Cancer Cells

AMPK and the mTORC1 complex are two main regulators of autophagy [50]. Thus, we next sought to investigate whether these two factors were involved in the induction of autophagy mediated by ^V600E^BRAF inhibition.

**Figure 2:**
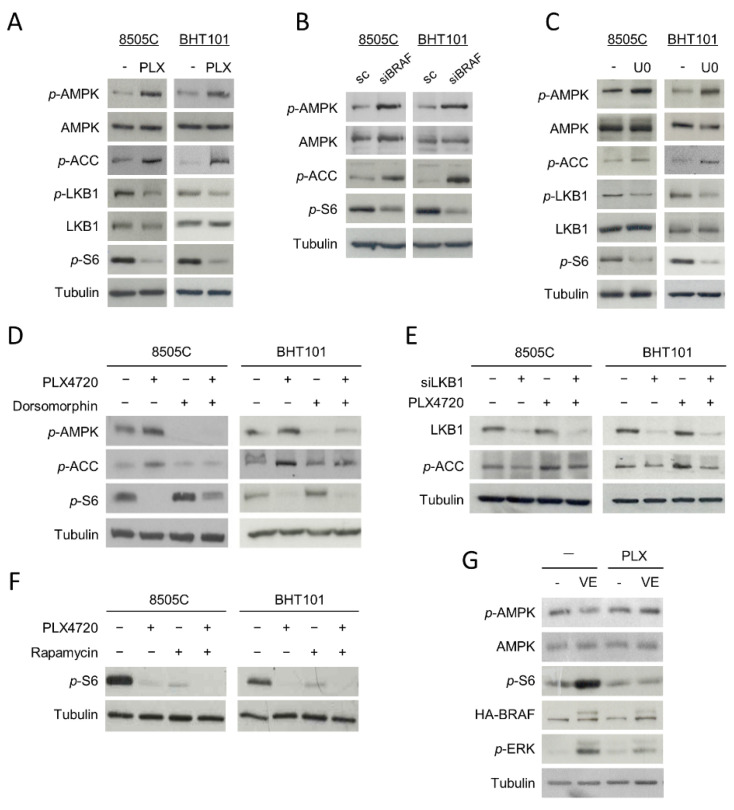
Inhibition of ^V600E^BRAF activates the AMPK pathway and inhibits mTOR in thyroid cancer cells. Phosphorylation levels of AMPK (*p*-AMPK), ACC (*p*-ACC), LKB1 (*p*-LKB1) and S6 (*p*-S6), together with total protein levels of AMPK and LKB1 in 8505C and BHT101 cells treated with DMSO (-) or PLX4720 (PLX) (**A**) or U0126 (U0) (**C**) for 24 h. (**B**) *p*-AMPK, *p*-ACC, *p*-S6 and total AMPK levels in cells transfected with a scrambled oligo control (sc) or a specific siRNA for BRAF (siBRAF) for 72 h. (**D**) *p*-AMPK, *p*-ACC and *p*-S6 expression levels in cells incubated with DMSO or PLX4720, alone or with Dorsomorphin, for 24 h. (**E**) Expression of LKB1 and *p*-ACC in cells transfected with an oligo control or specific siRNA for LKB1 (siLKB1) for 48 h, and then incubated in the absence or presence of PLX4720 for 24 h. (**F**) *p*-S6 levels in cells incubated with DMSO or PLX4720, in the absence or presence of Rapamycin, for 24 h. (**G**) Levels of *p*-AMPK, AMPK, *p*-S6, HA-BRAF and phosphorylated ERK (*p*-ERK) in both WRO-mock (-) and WRO-VE (VE) cells treated with DMSO or PLX4720 for 24 h. For each experiment, membranes were reprobed with anti-β-Tubulin as a loading control. Blots are representative of experiments performed three times with similar results.

Since AMPK is activated by phosphorylation at residue Thr172, we first studied whether the phosphorylation status of this residue was affected by ^V600E^BRAF inhibition. Interestingly, an increase in the phosphorylation levels of the Thr172 of AMPK in PLX4720-treated cells was observed (Figure 2A). In parallel, the phosphorylation level of its substrate Acetyl-CoA carboxylase (ACC) was higher in cells treated with PLX4720 compared to control cells, indicating that ^V600E^BRAF inhibition induces activation of AMPK (Figure 2A). Similar results were obtained in ^V600E^BRAF-knockdown cells (Figure 2B) and in cells treated with the MEK inhibitor U0126 (Figure 2C), suggesting that ^V600E^BRAF is regulating AMPK activity through the MEK-ERK pathway. Furthermore, these results were confirmed by analysing the phosphorylation levels of AMPK and ACC in the absence or presence of the selective AMPK inhibitor, Dorsomorphin. Our data show that this compound decreased the phosphorylation levels of both AMPK and ACC in basal conditions and blocked the increased phosphorylation of these proteins induced by ^V600E^BRAF inhibition (Figure 2D).

The LKB1 kinase directly activates AMPK by phosphorylating it at Thr172 residue and it is inhibited by phosphorylation at Ser428 residue through the ERK pathway. Then, we addressed whether the mechanism by which ^V600E^BRAF inhibition activates AMPK was dependent on LKB1 in 8505C and BHT101 cells. Our results revealed a decrease in the phosphorylation of LKB1 at Ser428 in PLX4720-treated cells, indicative of an increase in its kinase activity, parallel to the higher phosphorylation of AMPK at Thr172 observed (Figure 2A). Consistently, similar effects were obtained after treatment with the MEK inhibitor, U0126 (Figure 2C). To confirm that the activation of AMPK produced by ^V600E^BRAF inhibition was due to an increase in LKB1 kinase activity, we abrogated the expression of LKB1 with specific siRNA followed by the assessment of AMPK activation by measuring the phosphorylation of its substrate ACC (Figure 2E). As expected, the lack of the expression of LKB1 decreased both the basal and PLX4720-induced phosphorylation of ACC in both cell lines (Figure 2E). All these data indicate that AMPK activation following ^V600E^BRAF inhibition is mediated by LKB1 through a MEK-ERK dependent mechanism.

On the other hand, it has been previously reported that the RAF-MEK-ERK pathway activates mTORC1 complex in different models. In addition, as mentioned before, mTORC1 complex is a main regulator of autophagy. Therefore, we next analysed the effect of ^V600E^BRAF inhibition on the activation of this complex in 8505C and BHT101 cells by measuring the activity of the kinase mTOR, which forms the catalytic subunit of this complex. A significant decrease in the phosphorylation levels of mTOR effector, S6, was observed following treatment with PLX4720 (Figure 2A). Similar effects were obtained in BRAF-knockdown cells (Figure 2B) and in U0126-treated cells (Figure 2C), indicating that mTOR activation is dependent on ^V600E^BRAF. These results were confirmed by treating our cells with the inhibitor of mTOR, Rapamycin. Individual treatment with Rapamycin or PLX4720 decreased S6 phosphorylation in both cell lines, while a higher decrease was observed after combined treatment with both inhibitors (Figure 2F).

As AMPK downregulates mTORC1 in different kinds of tumours, we also studied the role of AMPK on ^V600E^BRAF-induced mTOR activation. For this purpose, we assessed mTOR activity in cells treated with Dorsomorphin, in the absence or presence of PLX4720. Interestingly, Dorsomorphin treatment increased the phosphorylation levels of S6 in basal conditions and partially reversed the PLX4720-mediated reduction of S6 phosphorylation (Figure 2D). These results demonstrate that ^V600E^BRAF inhibition abrogates the activation of mTOR, at least in part through AMPK activation.

To further substantiate these findings, we measured both AMPK and mTOR activity in the WRO cell line, in which we stably overexpressed ^V600E^BRAF by lentiviral infection [12]. The overexpression of ^V600E^BRAF led to an increase in the phosphorylation levels of its downstream target, ERK (Figure 2G). Consistent with our previous results, a significant reduction in the phosphorylation of AMPK and an increase in mTOR activity was observed in WRO-VE cells, when compared to control WRO-mock cells (Figure 2G). In addition, treatment with PLX4720 reversed these effects in WRO-VE cells, without affecting them in WRO-mock control cells (Figure 2G).

All these data clearly demonstrate that ^V600E^BRAF regulates AMPK and mTOR activities in thyroid cancer cells.

### 2.3. ^V600E^BRAF Inhibition Induces Autophagy through AMPK-ULK1 Activation in Thyroid Cancer Cells

Several studies have reported that autophagy is activated by AMPK and inhibited by mTOR through ULK1 regulation. ULK1 is an essential part of the initiation complex of autophagy and its phosphorylation at Ser555 is necessary to start the autophagic flux. Hence, to further investigate the molecular mechanism of autophagy induction by ^V600E^BRAF inhibition we analysed the phosphorylation status of ULK1 at Ser555, as well as the LC3-II/LC3-I ratio and p62 protein levels in 8505C and BHT101 cells treated with PLX4720 alone or in combination with either Dorsomorphin or Rapamycin.

**Figure 3:**
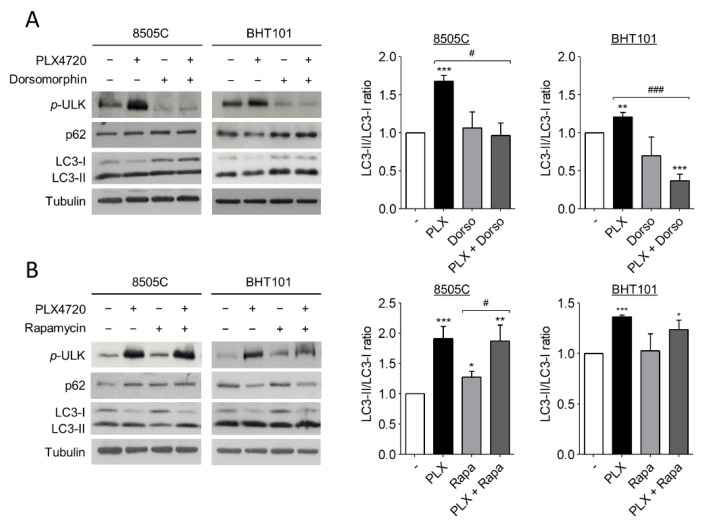
^V600E^BRAF inhibition increases autophagy through AMPK-ULK1 signalling. Expression levels of phosphorylated ULK1 at Ser555 (*p*-ULK), p62 and LC3 (left blots), and quantitative analysis of LC3-II/LC3-I ratios (right graphs) in 8505C and BHT101 cells treated with DMSO (-) or PLX4720 (PLX), in the absence or presence of Dorsomorphin (Dorso) (**A**) or Rapamycin (Rapa) (**B**) for 24 h. For each experiment, membranes were reprobed with anti-β-Tubulin as a loading control. Graphic bars represent the LC3-II/LC3-I ratio, calculated after quantitation of LC3-II and LC3-I bands of the blots, and are presented as fold induction relative to the untreated cells. Blots are from one representative experiment and data shown represent the mean ± SEM of the quantitation of at least three independent experiments performed with similar results. Significant differences compared to the corresponding controls: * 0.01 < *p* < 0.05, ** 0.001 < *p* < 0.01, *** *p* < 0.001, compared to untreated cells; ^#^ 0.01 < *p* < 0.05, ^###^ *p* < 0.001, compared to Dorsomorphin or Rapamycin, respectively.

Remarkably, the degree of ULK1 phosphorylation at Ser555 were higher in PLX4720-treated cells than in control cells, indicating an increase in ULK1 activation following V600EBRAF inhibition (Figure 3A,B). Additionally, the presence of Dorsomorphin reduced the basal levels of phosphorylated ULK1 and, interestingly, reversed the increased levels of ULK phosphorylation achieved by PLX4720 treatment (Figure 3A), demonstrating that AMPK activates the initiation complex during autophagy. By contrast, the inhibition of mTOR by Rapamycin did not exert any effect under all conditions analysed (Figure 3B). These results suggest that V600EBRAF inhibition increases ULK1 phosphorylation through AMPK activation. Regarding LC3-II/LC3-I ratio, Dorsomorphin did not affect basal levels in 8505C andBHT101 cells, but this AMPK inhibitor reduced the increase in the ratio achieved by the single treatment with PLX4720 (Figure 3A). Accordingly, Dorsomorphin also reversed the decrease in p62 levels observed after ^V600E^BRAF inhibition in BHT101 cells (Figure 3A). However, similar to the results obtained when ULK1 phosphorylation was analysed, mTOR inhibition with Rapamycin did not show any significant difference in both LC3-II/LC3-I ratio and p62 levels, neither in basal conditions nor in PLX4720-treated cells (Figure 3B).

These data demonstrate that ^V600E^BRAF inhibition induces autophagy through ULK1 activation in thyroid cancer cells. Importantly, they also reveal that this mechanism is dependent on AMPK activation but not on mTOR.

### 2.4. Autophagy Blockage Decreases Cell Viability and Sensitizes Thyroid Cancer Cells to ^V600E^BRAF Inhibition

Autophagy has a cytoprotective role under therapeutically stress conditions in different types of cancer. Given that the reduction of ^V600E^BRAF activity induces this process, we next investigated the role of autophagy on cell survival in response to ^V600E^BRAF inhibition. For this purpose, thyroid cancer cells were treated with a combination of PLX4720 with either Bafilomycin A1 or Chloroquine, two well-known autophagy inhibitors.

**Figure 4:**
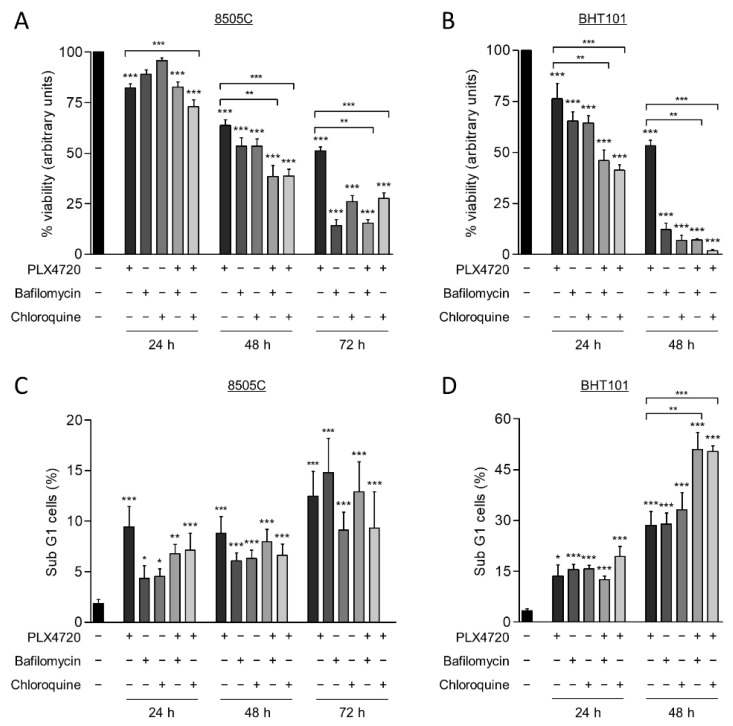
Autophagy blockage induces cell death and sensitize thyroid cancer cells to ^V600E^BRAF inhibition. Cell viability measured by MTT assay in 8505C (**A**) and BHT101 (**B**) cells following 24, 48, or 72 h treatment with PLX4720, Bafilomycin A1 or Chloroquine alone, or with PLX4720 in combination with each of the autophagy inhibitors. Results shown are the mean ± SEM of three independent experiments performed in triplicate. Apoptosis was measured in 8505C (**C**,**D**) BHT101 cells treated as in (**A**,**B**), respectively, by quantitation of the sub-G1 fractions of PI-stained cells by flow cytometry. Results shown are the mean ± SEM of three independent experiments performed in duplicate. Significant differences compared to the corresponding controls: * 0.01 < *p* < 0.05, ** 0.001 < *p* < 0.01, *** *p* < 0.001.

As expected, treatment with PLX4720, as a single agent, exerted a time-dependent reduction of cell viability in both cell lines, 8505C and BHT101 (Figure 4A,B). Similarly, both Bafilomycin A1 and Chloroquine also decreased cell viability in a time-dependent manner in both cell lines, although the effect was much greater in BHT101 cells than in 8505C cells. Accordingly, both autophagy inhibitors triggered a decrease in BHT101 cell viability of approximately 90% after 48 h treatment, whereas 8505C cells reached similar reduction after 72h treatment with each inhibitor (Figure 4A,B). Notably, we also observed that the effects on cell viability produced by the combination of autophagy and ^V600E^BRAF inhibition were greater than the effects triggered by individual treatments. Again, higher sensitivity to these combinations was achieved in BHT101 cells than in 8505C cells (Figure 4A,B). All these results indicate that autophagy has a cytoprotective role in these thyroid cancer cells and that the inhibition of this process significantly enhances the cytotoxic efficacy of ^V600E^BRAF inhibition.

To gain further insight into the mechanism involved in the reduction of cell viability produced by the combination of the autophagy inhibitors and PLX4720, apoptosis was determined in the thyroid cancer cells by analysing the percentage of cell population with subG1 DNA content. For this purpose, identical conditions and treatments as in the previous assay were used. These experiments revealed that, although PLX4720, the autophagy inhibitors and combined treatments induced apoptosis in 8505C cells at all times assayed, no significant differences between these conditions were observed (Figure 4C). However, BHT101 cells treated with the combination of autophagy and ^V600E^BRAF inhibitors displayed a significant increase in apoptosis compared to cells treated with single agents, as evidenced by the increased levels of subG1 population achieved at 48 h (Figure 4D). Altogether, these findings demonstrate that targeting autophagy enhances the cell death induced by ^V600E^BRAF inhibition and may overcome the resistance to BRAFi in thyroid cancer cells.

### 2.5. Targeting Autophagy Enhances Antitumor Effect of PLX4720 In Vivo

**Figure 5:**
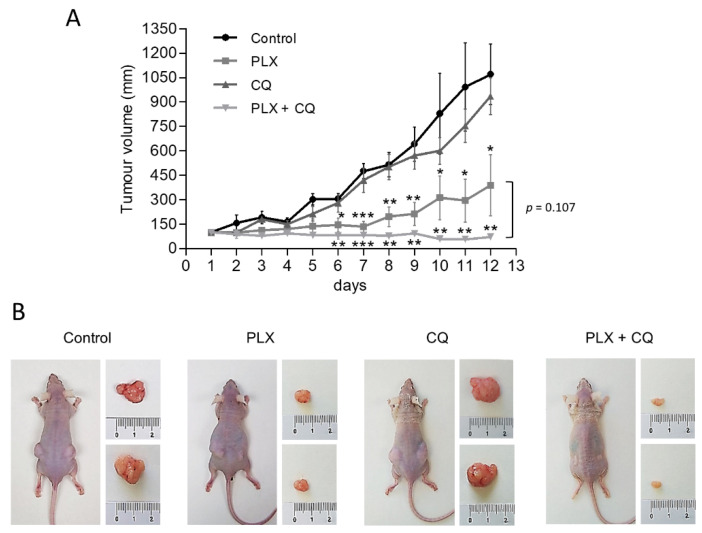
Autophagy inhibition enhances antitumor activity of PLX4720 BRAFi in vivo. Mice bearing subcutaneous BHT101 cells-derived xenografts on both flanks were randomly divided into four groups and treated intraperitoneal with vehicle (control), 25 mg/kg/day PLX4720 (PLX), 60 mg/kg Chloroquine (CQ) or combination of both (PLX + CQ) for 12 days. (**A**) Curves of tumour growth monitored by measuring the tumour volumes over time after the treatments. Data are shown as the mean ± SEM; *n* = 6. (**B**) Representative images showing mice from different treatment groups with tumours on day 12 of treatment. Significant differences compared to the corresponding untreated control: * 0.01 < *p* < 0.05, ** 0.001 < *p* < 0.01, *** *p* < 0.001.

To further assess the preclinical relevance of our findings and corroborate the therapeutic benefit of inhibiting both autophagy and ^V600E^BRAF in thyroid cancer, we conducted in vivo experiments assessing the growth of BHT101-derived subcutaneous xenograft tumours in athymic nude mice. After ensuring equivalent and sufficient tumour growth, animals were randomly assigned to four groups and treated with vehicle only, PLX4720, Chloroquine or a combination of both drugs. Importantly, in all experimental conditions, no signs of toxicity or changes in body weight were found (data not shown), confirming a good tolerability of all treatments. Regarding monitoring the antitumor efficiency of our treatments, Chloroquine alone had moderate effects on tumour growth, whereas PLX4720-treated mice displayed a significant reduction in the size of the tumours (Figure 5). Interestingly, mice treated with the combination of Chloroquine and PLX4720 showed a greater reduction in tumour growth, resulting in significant differences when compared to animals subjected to single treatments with Chloroquine or PLX4720. Together, these findings demonstrate that autophagy inhibition can potently enhance the in vivo anti-tumorigenic effects of ^V600E^BRAF inhibition with PLX4720 in thyroid cancer.

## 3. Discussion

Although several studies have described the cytoprotective role of autophagy in cancer cells, the molecular mechanisms involved in the activation of autophagy in ^V600E^BRAF-positive thyroid cancer cells and its implication in the resistance to inhibition of ^V600E^BRAF remain largely unknown. Here, we provide new insights into these mechanisms and demonstrate that autophagy is essential for survival of thyroid cancer cells after specific treatment with the BRAFi, PLX4720.

Firstly, we reveal that both ^V600E^BRAF inhibition with PLX4720 and abrogation of its expression induce autophagy in thyroid tumour cells. This is shown by the increased LC3-II/LC3-I ratio and the enhanced autophagic flux observed in the presence of Bafilomycin A1 in both 8505C and BHT101 cell lines. In concordance, ^V600E^BRAF inhibition induces p62 degradation in BHT101 cells. Although p62 reduction in 8505C cells was not observed, we confirmed that ^V600E^BRAF inhibition induces autophagy in this cell line by the increase in the number of autophagosomes and autolysosomes observed after PLX4720 treatment. This difference indicates that changes in p62 levels could be cell type dependent. Thus, it could be possible that the degradation times of this protein are different between cell lines. Alternatively, in 8505 C cells, p62 might have important roles in other cellular processes, regardless of autophagy induction by ^V600E^BRAF inhibition. Hence, 8505C cells would need to prevent its autophagy-dependent degradation in this context [51,52]. Our results are in agreement with those showing induction of autophagy by ^V600E^BRAF inhibition in other cell types. For instance, increased levels of autophagy have been observed in melanoma cell lines and biopsies from ^V600E^BRAF melanoma patients treated with PLX4720 compared to their respective non-treated controls [40]. Moreover, since the treatment with the BRAFi Vemurafenib also enhances autophagy in melanoma [40,41], colorectal cancer [47] and thyroid cancer cells [46], our results further reinforce the fact that induction of autophagy by ^V600E^BRAF inhibition seems to be an extended effect in different types of cancer, in which this oncogene plays an important role.

Our study also demonstrate that ^V600E^BRAF inhibition increases autophagy flux through an AMPK dependent mechanism. We show that both ^V600E^BRAF inhibition and knockdown increases AMPK activity through the MEK-ERK pathway. Furthermore, we describe that AMPK activation following ^V600E^BRAF inhibition is dependent on its upstream activator LKB1. These results are further supported by the reduction on AMPK activity observed after ^V600E^BRAF overexpression in ^WT^BRAF thyroid tumour cells. In agreement with this, it has been reported that the inhibition of ^V600E^BRAF in melanoma increases LKB1 activity that, in turn, stimulates AMPK [53]. Similarly, it has also been shown that colorectal cancer cells display an increase in AMPK activity after treatment with either PLX4720 or Vemurafenib [47]. However, to our knowledge, this is the first study that demonstrates the negative regulation of AMPK by ^V600E^BRAF in thyroid cancer cells.

On the other hand, AMPK is involved in mTORC1 inhibition [54] and this complex is one of the main regulators of autophagy. In our model, mTOR activation is dependent on ^V600E^BRAF-MEK-ERK pathway, as observed in other cell types [55,56], but it was also partially mediated by AMPK. This is supported by the inverse correlation between AMPK and mTOR activities after inhibition of ^V600E^BRAF, and by the fact that AMPK inactivation with Dorsomorphin increases basal mTOR activity and partially recovers the inhibition achieved by PLX4720 treatment. Therefore, we demonstrate that ^V600E^BRAF activates mTOR through the canonical MEK-ERK-p90RSK-TSC2 pathway, but also through AMPK inhibition. Interestingly, this dual effect has also been shown in melanoma cells, where abrogation of LKB1 expression cooperates with ^V600E^BRAF to activate mTOR [57].

We also demonstrate that autophagy is mediated by AMPK-stimulated ULK1 activation. In this sense, it is known that AMPK activates autophagy by phosphorylation of ULK1 in several residues, including Ser555 [50,58]. Conversely, although it is well known that autophagy is inhibited by mTOR, we did not observe any changes in ULK1 phosphorylation and autophagy levels in Rapamycin-treated cells, in neither basal nor PLX4720-activated conditions. Thus, we conclude that mTOR is not involved in PLX4720-induced autophagy and it is plausible that ^V600E^BRAF inhibition bypasses mTORC1 complex to enhance autophagy in thyroid tumour cells with this mutation. In this sense, one possible mechanism is that AMPK directly activates ULK1 after PLX4720 treatment. This hypothesis is in agreement with other results reported in melanoma and colorectal cancer cells, showing that Vemurafenib treatment was followed by an increase in AMPK activity and high levels of activated ULK1, which in turn, were correlated with autophagy induction [47,59]. In addition, the inhibition of the downstream ^V600E^BRAF target, MEK, also activates autophagy through the LKB1-AMPK-ULK1 axis in pancreatic ductal carcinoma [60]. Of note, given that autophagy is regulated by multiple factors and at different levels, we cannot discard alternative mechanisms involved in PLX4720-mediated autophagy induction. In line with this, Vemurafenib has shown to enhance autophagy through an increased ER stress response by a MEK/ERK-independent mechanism in thyroid tumour cells [46]. However, the role of ER stress is controversial since it has been observed that both the activation and inhibition of ^V600E^BRAF induce this response in melanoma, leading to an increase in cellular autophagy [40,61]. In this sense, although we have not studied the effects of PLX4720 on ER stress response, our results point to a MEK-ERK dependent regulation of autophagy produced by ^V600E^BRAF inhibition. Thus, we can speculate that ^V600E^BRAF is modulating autophagy through both MEK-ERK dependent and independent mechanisms in thyroid cancer cells, the former being responsible for AMPK activation.

Contradictory results have been reported regarding the cytoprotective role of autophagy in cancer cells harbouring the ^V600E^BRAF mutation [39,41,47,61,62,63], including thyroid cancer cells [43,44,45,46,64]. However, our study clearly demonstrate that autophagy inhibition sensitizes thyroid cancer cells to specific inhibition of ^V600E^BRAF. This is demonstrated by the decrease in the cell survival and the induction of apoptosis achieved by combined treatments of PLX4720 and Bafilomycin A1 or Chloroquine. Additionally, these results are further supported by the in vivo experiments, in which tumour growth is significantly halted by the combination of PLX4720 and Chloroquine. In this regard, the differences observed between cell lines might be due to a higher basal autophagy levels of the BHT101 cells, which lead to more dependency on this process for surviving. Our data are similar to those obtained in other types of cancer such as melanoma, brain and colorectal cancer [40,43,47], and more specifically to recent studies in thyroid cancer cells showing that different antitumorogenic compounds activate autophagy and that inhibition of this process enhances their anticancer effects [46,62,63,64,65,66,67].

Our study provides novel insights into the mechanisms whereby ^V600E^BRAF inhibition activates autophagy through the induction of the LKB1-AMPK-ULK1 pathway. Moreover, we reported that autophagy has a cytoprotective role and its blockage potentiates PLX4720-induced cell death of thyroid cancer cells carrying ^V600E^BRAF mutation, both in vitro and in vivo. Therefore, this study provides a rational to develop therapeutic strategies targeting the AMPK pathway and/or autophagy that can contribute to enhance the efficacy of ^V600E^BRAF inhibitors and to overcome the acquired resistance to these drugs in thyroid cancer.

## 4. Materials and Methods

### 4.1. Cell Lines, In Vitro Treatments, Plasmids, siRNA and Transfections

Human ATC-derived cell lines 8505C and BHT101, both harbouring ^V600E^BRAF mutation, were purchased from the German Collection of Microorganisms and Cell Cultures (DSMZ, Braunschweig, Germany) WRO-mock (control) and WRO-VE (stably overexpressing ^V600E^BRAF) cells were generated by lentiviral infection of the follicular thyroid cancer (FTC) derived cell line WRO, that harbour ^wt^BRAF, as described in Baquero et al. [12]. All cell lines were cultured in DMEM supplemented with 10% foetal bovine serum (FBS) and 1% penicillin/streptomycin at 37 °C in a 5% CO_2_ atmosphere. Identity was confirmed vs. published data [12], using standard sequencing techniques.

For in vitro treatments, the cells were incubated for the indicated times with either vehicle (dimethyl sulfoxide, DMSO), 10 μM PLX4720 (Axon MedChem, Groningen, The Netherlands), 10 μM U0126 (Promega, Madison, WI, USA), 10 μM Dorsomorphin (Tocris Bristol, UK), 100 nM Bafilomycin A1, 50 nM Rapamycin or 10 μM Chloroquine (Sigma, St. Louis, MO, USA).

For siRNA experiments, the cells were transfected using LipofectAMINE (Invitrogen, Life Technologies, Carlsbad, CA, USA), according to manufacturer’s protocols in 1 mL of OPTIMEM medium with 100 nM BRAF (5′-CAGUCUACAAGGGAAAGUG-3′), ATG5 (5′-GGAUGCAAUUGAAGUCAU-3′), LKB1 (5′-AGGAGGUUACGGCACAAAA-3′), or Silencer^TM^ negative control#1 specific siRNA (Ambion, Carlsbad, CA, USA). After 6 h incubation with the RNA-complex, the medium was replaced with 2 mL of fresh medium containing 10% FBS and cells were treated at the indicated times, as stated in figure legends.

The expression vector ptfLC3, encoding rat LC3 fused to mRFP and EGFP, was a gift from Tamotsu Yoshimori (Addgene plasmid#21074; http://n2t.net/addgene:21074 (accessed on 5 May 2021) RRID: Addgene_21074) [43]. First, 1 μg vector was transfected using LipofectAMINE and OPTIMEN as described above. Next, the cells were incubated for 6 h in the presence of the DNA:lipofectAMINE complexes, and then washed and maintained in complete medium for 48 h until treatments.

### 4.2. Cell Lysis and Western Blot Analysis

Total cell extracts preparation and Western blot analysis were performed as previously described [13]. The antibodies used were anti-BRAF and anti-AMPK (Santa Cruz Biotechnology, Dallas, TX, USA); anti-p62 (BD Biosciences, Franklin Lakes, NJ, USA); anti-ATG5, anti-phospho-LKB1 (S428), anti-LKB1, anti-phospho-ACC (S79), anti-phospho-AMPK (T172), anti-phosho-S6 (S235/236) and anti-phospho-ULK (S555) (Cell Signalling Technology, Danvers, MA, USA); anti-LC3, anti-phospho-ERK and anti-β-tubulin (Sigma, St. Louis, MO, USA); and peroxidase-conjugated secondary antibodies (DAKO, Glostrup, Denmark). For LC3-II/LC3-I ratio calculation, data densitometry of blots from different experiments was used to quantify the protein bands.

### 4.3. Immunofluorescence Assays

The cells were seeded in µ-imaging dish 35 mm (ibidi GmbH, Germany) and transfected with the plasmid ptfLC3. Then, 48 h after transfection, the cells were treated as stated in figure legends. Before the analysis, the medium was aspirated, the cells were washed with phosphate-buffered saline (PBS) and the nuclei were stained with 0.2 µg/mL Hoechst dye (Thermofisher Scientific, Waltham, MA, USA) in PBS for 10 min. After this, the cells were washed twice and left in PBS. Fluorescence was visualized in a confocal microscope Leica TCS-SP5 (Leica microsystem, Wetzlar, Germany) by ICTS “NAMBIOSIS”, specifically by the Confocal Microscopy Service: CIBER-BNN at the UAH (CAI Medicine Biology).

### 4.4. Cell Viability Assays

Cell viability was assessed at different times with MTT (**3**-(**4**,**5**-Dimethythiazol–**2**-yl)-(**2**,**5**-diphenyltetrazolium) bromide) assay. After treatments, MTT was added (final concentration 0.5 mg/mL) and cells were incubated for 3 h. Formazan crystals were dissolved and absorbance measured at 570 nm in microplate reader FL600 (BioTek, Winooski, VT, USA). Each experimental group had three duplicate wells.

### 4.5. Quantification of Sub-G1 DNA Content by Flow Cytometry

Apoptosis was quantified measuring the fraction of sub-G1 DNA content cell population stained with propidium iodide (PI) by flow cytometry analysis. After treatments, adherent and floating cells were collected, washed with PB, and fixed with 70% ethanol. The fixed cells were washed twice with PBS and treated with RNase (1 mg/mL). Cellular DNA was stained with 5 ng/mL PI in PBS and cells were analysed on a FACScan flow cytometer (Becton Dickinson, Franklin Lakes, NJ, USA). Percentages of cells in different cell cycle phases were calculated from DNA histograms. Cells with sub-G_1_ DNA content were considered apoptotic.

### 4.6. Xenograft Models

All animal experiments were carried out with the approval of the Ethical Committees of the University of Alcalá and the Comunidad de Madrid (UAH-CAM, procedure PROEX 178/17), in accordance with the Spanish institutional regulation (RD 53/2013) for the housing, care and use of experimental animals and met the European Community directives regulating animal research. Athymic nude-Foxn1 (nu/nu) mice (aged 4 weeks) were purchased from Envigo RMS (Barcelona, Spain) and housed under pathogen-free conditions with access to food and water ad libitum. BHT101 cells (5 × 10^6^ in 200 mL of in serum-free DMEM medium) were injected subcutaneously into both flanks of the mice. When tumours reached a volume of 70 mm^3^, the mice were randomly divided into four experimental groups (*n* = 6 in each group). The six mice in each group were administered interperitoneally for 12 days with DMSO, PLX4720 (25 mg/kg/day), Chloroquine (CQ) (60 mg/kg/day), or a combination of PLX4720 and CQ. The tumour size was measured every day with a digital callipers and volume calculated as (length × width^2^)/2. At the end of the study, the mice were euthanized by placing them in a CO_2_ gas-filled chamber, and the tumours were dissected, weighed and photographed.

### 4.7. Statistical Analysis

All statistical analyses were performed with GraphPad Prism v.8.1.1 (GraphPad Software, San Diego, CA, USA). Differences between groups were assessed using the unpaired Student’s *t*-test *p*-values indicating significant differences are shown in the figures as follows: *** *p* < 0.001, ** *p* < 0.01, * *p* < 0.05.

## Data Availability

The data presented in this study are available within the article text and figures. Data are available from the corresponding author upon request.
